# Analysis of Early-Age Hydration Behavior and Micro-Mechanism of Coral Sand Cement Mortar

**DOI:** 10.3390/ma15031074

**Published:** 2022-01-29

**Authors:** Yue Qin, Fanhua Meng, Zhao Zhang

**Affiliations:** 1 School of Civil Engineering and Architecture, Wuhan University of Technology, Wuhan 430070, China; yqin@whut.edu.cn; 2 Department of Geotechnical Engineering, Dalian University of Technology, Dalian 116024, China; zz110zz@mail.dlut.edu.cn

**Keywords:** coral sand cement, Fiber Bragg Grating sensors, early-age, hydration behavior, proportion

## Abstract

Coral sand cement (CSC) mortar is increasingly used in reef projects, which is prepared by mixing coral sand with cement and water in certain proportions. Considering that early-age hydration behavior is closely related to the strength and durability of the mortar, the early-age hydration process and micro-morphology of CSC mortars with various water–cement ratios (W/C) and sand–cement ratios (S/C) were studied. A monitoring system based on FBG is proposed in this paper, which uses the high sensitivity and conformability of optical fiber to measure the hydration temperature and internal shrinkage strain simultaneously and continuously. The standard sand cement (SSC) mortar with the same sand gradation and mix proportion is also prepared for comparison. The micro-morphology is observed by a scanning electron microscope (SEM) for measurement results’ explanation. The results show that the variation of the hydration temperature and shrinkage strain with hydration time of both CSC mortars and SSC mortars follow a unimodal function. Differently, the peak hydration temperature for CSC is obviously lower than that of SSC. The peak temperature of CSC mortar decreases linearly with the increase in S/C, and the decrease rate of the peak temperature is higher for CSC with small W/C than that with higher W/C. For mortars with lower W/C, the peak shrinkage strain of CSC is larger than that of SSC. Meanwhile, for mortars with higher W/C, the peak shrinkage strain of CSC changes to be lower than that of SSC, which is attributed to the significant water absorption characteristic of CSC. Therefore, as an eco-friendly lightweight aggregate, CS is more suitable than SS for the design of high W/C and alleviating the hydration heat of mass concrete under the meeting of strength.

## 1. Introduction

With the rapid development of the ocean economy, offshore man-made islands have been widely constructed recently [1,2], which has led to a surge in demand for cementitious materials [3]. Nevertheless, traditional construction materials are not locally available, and the transportation and modification of ordinary concrete will cause problems of high construction costs and great environmental damage. Therefore, the use of sea sand as an alternative to manufacturing concretes has been widely studied. Among various sea sands, coral sand (CS) is widely existing and available [4,5,6]. However, CS has many limitations for cementitious materials, such as a lower density, higher porosity, and water absorption compared with ordinary aggregates [7].

Since Howdyshell [8] first evaluated the workability of developing concrete with coral aggregate, studies have increasingly concentrated on the mixture design, mechanical properties, and durability characteristics of coral concrete. Coral concrete is commonly classified as a lightweight aggregate concrete due to the similar mechanical properties as ordinary lightweight aggregate [9,10]. Arumugam et al. [7] investigated the strength development law of coral concrete and found that strength grew rapidly in the early stage but slowly in the later stage. Wang et al. [2] observed enhanced strength caused by the cement products interlocked in pores of the coral sand. Sun [11] studied the mechanical properties of coral concrete corresponding to the different proportions of silica fume and found that a 20–30% substitution rate can effectively improve the compressive and splitting tensile strength. Da et al. [12] obtained the whole stress–strain curve ascent stage of coral concrete through prismatic specimens under uniaxial compression and found it was rapidly damaged after peak strain. Mineral admixtures such as fly ash, blast furnace slag, and metakaolin are proven to effectively improve the compressive strength, frost resistance, and durability of coral concrete [13,14,15]. Pitiwat et al. [16] investigated the durability of coral concrete based on the Cl-diffusivity and suggested that the chloride penetration resistance was higher than the ordinary one.

Relevant research mainly focuses on the later strength and shrinkage strain of coral concrete. However, the early-age hydration behavior of CSC, usually referring to the first 48 h, has significant effects on the long-term mechanical properties of concrete [17,18]. The hydration process of cement has been measured by a combination of X-ray computed tomography, scanning electron microscopy (SEM), and electromechanical impedance (EMI) [19,20,21,22,23,24]. Though these methods can offer accurate analysis, they can hardly provide real-time monitoring and intuitive shrinkage results. Moreover, the early-age shrinkage of concrete is difficult to obtain due to a lack of measurement techniques for unformed concrete [25]. With the popularization of Fiber Bragg Grating (FBG) in structural health monitoring [26,27], its advantages of high precision, deformation coordination, small volume, long durability, and intuitive measurement results have been widely accepted by researchers and it has been successfully applied in monitoring hydration temperatures and the early-age shrinkage of concrete [28,29].

Various studies have shown that the water–cement ratio (W/C) and sand–cement ratio (S/C) are two important factors affecting the hydration and later mechanical characteristics of standard sand cement (SSC) mortar [30,31,32,33]. Meanwhile, for CSC mortar, the effects of W/C and S/C on the early-age hydration behavior are still uncertain. Accordingly, the effect of the proportion of CSC (i.e., W/C and S/C) on the early-age (7-days) hydration behavior and micro-morphology is investigated in this study. The hydration temperature and internal shrinkage strain of CSC mortars are measured through a Fiber Bragg Grating (FBG) monitoring system and the micro-morphology is observed by a scanning electron microscope (SEM). Parallel tests of SSC mortars with the same particle gradation and proportions are carried out for comparison.

## 2. Materials and Methods

### 2.1. Materials

**Figure 1:**
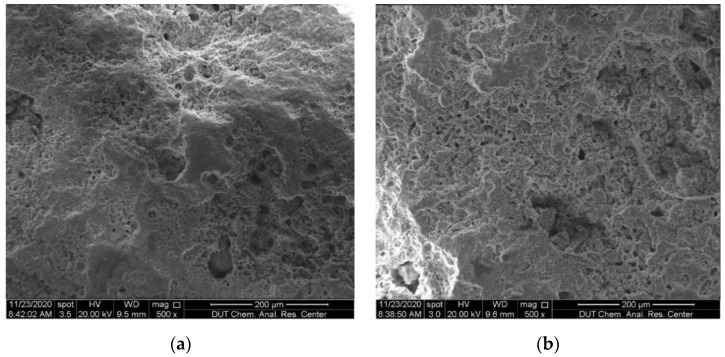
Typical micro-morphologies of coral sand observed by SEM with 500 magnifications: (**a**) Particle A; (**b**) particle B.

**Figure 2:**
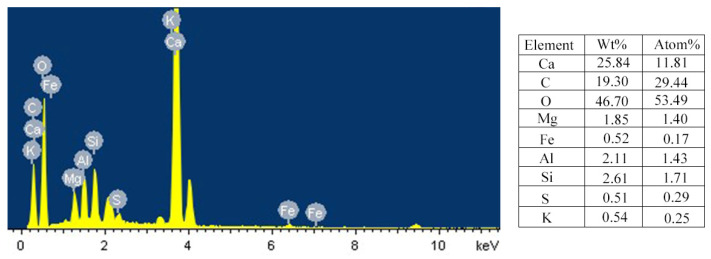
Element content of CSC sample.

**Figure 3:**
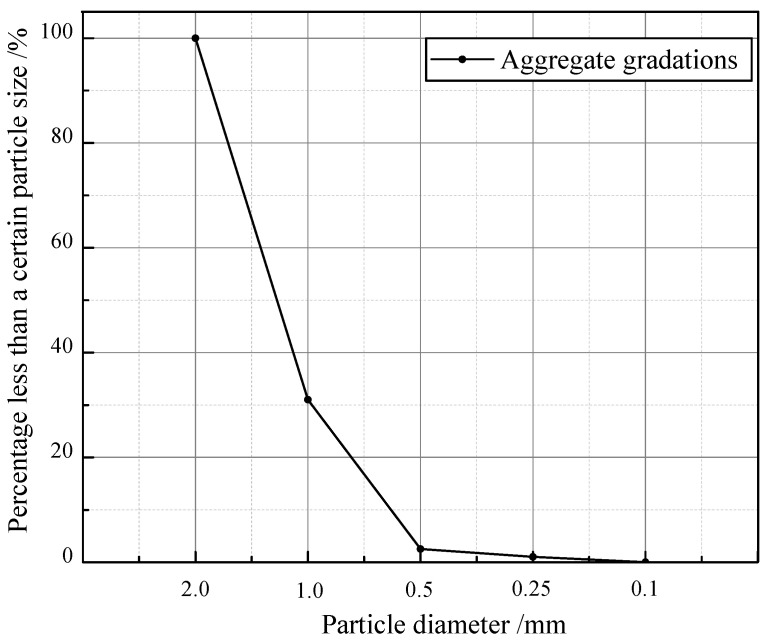
Particle grading of coral sand and standard sand in CSC and SSC.

The materials used in this study include coral sand, OPC 52.5 cement supplied by Zhucheng Yangchun Cement Co., Ltd. (Weifang, China), and standard sand supplied by China ISO Sand Co., Ltd. (Xiamen, China) The maximum particle size of coral sand was controlled at 2 mm by sieving to ensure uniformity of the slurry to better explore the early-hydration characteristics of cement mortar. The micro-morphology of CS observed by SEM is shown in Figure 1. It shows that as a naturally formed mineral material, CS has abundant internal pores. The densities of CS and SS in our paper are both the bulk density measured according to the Chinese standard for building sand GB/T 14684-2011. The density of CS passing through a 2 mm sieve is 2.78 g/cm^3^ [34], and the density of SS is measured as 2.79 g/cm^3^ according to the standard. The element content of CSC is shown in Figure 2, and the content of Ca, C, and O are found to be higher. This is because the chemical composition of CS mainly contains CaCO_3_, which exists in the form of microcrystalline calcite aggregate [2,35]. The particle grading of sand used in this study is shown in Figure 3, which is obtained through the sieving method according to the Standard for geotechnical testing method GB/T 50123-2019 with the standard sieve series (2 mm, 1 mm, 0.5 mm, 0.25 mm, 0.1 mm).

### 2.2. Method

In this study, the FBG sensors provided by Wuhan Yu Guang Technology Co., Ltd. in Wuhan China, were adopted to measure the internal temperature change and shrinkage strain development of CSC and SSC mortar specimens during the early-hydration process. The principle of FBG is that the reflection wavelength in the fiber grid changes as the temperature and strain variates. For a single-mode silica fiber, the relationship among the above variables is expressed as follows [36,37]:

(1) $$\frac{\Delta \lambda_{B}}{\lambda_{0}}=c_{\varepsilon }\Delta \varepsilon +c_{T}\Delta T$$

where $\lambda_{0}$ is the initial wavelength of the FBG; $c_{\varepsilon }$ and $c_{T}$ are the coefficients considering strain and temperature’s influence independently, which are constant for one specific FBG, and $c_{\varepsilon }=0.78\times 10^{-6}$, $c_{T}=6.67\times 10^{-6}/℃$ in this study. According to Equation (1), the shrinkage strain of cement paste can be calculated as:

(2) $$\Delta \varepsilon =\frac{1}{c_{\varepsilon }}\left(\frac{\Delta \lambda_{B}}{\lambda_{0}}-c_{T}\Delta T\right)$$

Therefore, through the reasonable optical fiber measurement strategy and temperature compensation algorithm, the temperature and strain of cement hydration could be measured simultaneously.

### 2.3. Experimental Procedure

In order to explore the early-hydration behavior of CSC mortar, a total of 16 specimens of different proportions were prepared. Though the S/C is often larger than 1 for strength studies, the main purpose of this paper is to explore the early-hydration heat and shrinkage strain of CSC mortar, rather than the strength. Lower S/C is helpful to better reflect this explored phenomenon. Moreover, to better keep warm and moisturized, and avoid the interference of environmental factors, we select a smaller test tube (with a height of 115 mm and a diameter of 30 mm) for the test. When the S/C is large, the optical fiber and copper tube are difficult to insert into the sample, which has an obvious impact on the test results. Therefore, both the W/C and S/C are selected to be less than 1 in our study to ensure the workability of cement and the convenience of test operation. The same proportions were prepared for the SSC mortar specimens for comparison and the details are listed in Table 1. The procedure of fabrication and monitoring is presented subsequently.

**Figure 4:**
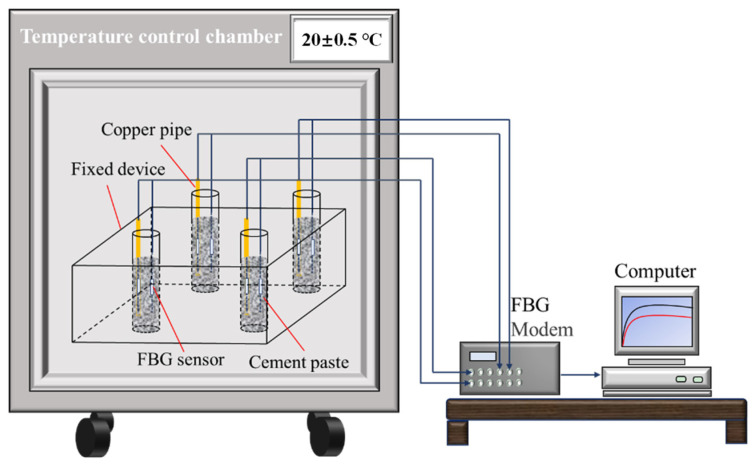
Schematic diagram of FBG monitoring system.

Before the experiment, to eliminate the influence of the material temperature on the hydration process and measurement results, the raw materials required for the test were put into a thermostat for at least four hours to keep the materials’ temperature at 20 °C. The specific aggregate and cement were weighted according to the mixture ratios and dry stirred in a beaker until fully mixed. Then the specific quality of tap water was added, and materials were stirred by a glass rod as soon as possible. Following this, the mixed cement paste was poured into the labeled test tubes. An FBG sensor and copper tube with another free FBG sensor inside were then inserted into the middle of the cement paste at the same time by slight vibrating. The diameter of the copper tube is 2 mm, which is small enough to avoid having a great impact on the hydration of cement slurry. Moreover, the slurry after mixing settled rapidly due to the small volume of mortar specimen, avoiding the influence of the fluidity of cement slurry on strain measurement. All the above operations are carried out in a temperature and humidity chamber to ensure a constant temperature and humidity environment. It should be noted that the addition of water would immediately lead to the hydration reaction, so it is necessary to ensure that the FBG modem is in the monitoring state in advance. In addition, the above operations need to be completed in a short time to ensure the successful measurement of the early hydration characteristic. Due to the high stiffness and thermal conductivity of the copper tube, the FBG inserted in copper is free of shrinkage strain and directly reflects the internal hydration temperature. By analyzing the monitoring results of the two FBGs according to Equation (2), the early hydration and shrinkage characteristics of the CSC mortar specimen can be obtained simultaneously. The schematic diagram of the FBG early-hydration and shrinkage characteristics monitoring system is shown in Figure 4. In this study, the test time fixes to 3500 min (about 2.5 days), which is sufficient to cover the early-hydration process according to the result from the pre-experiment.

## 3. Results and Discussions

The hydration temperature and shrinkage strain during the early hydration of CSC mortars were analyzed and the micro-morphology of the mortar specimens were observed. The parallel experimental analysis of SSC mortars was conducted as a comparison to discuss the early-hydration characteristic and micro-mechanism of CSC during early-hydration.

### 3.1. Early Hydration Temperature

**Figure 5:**
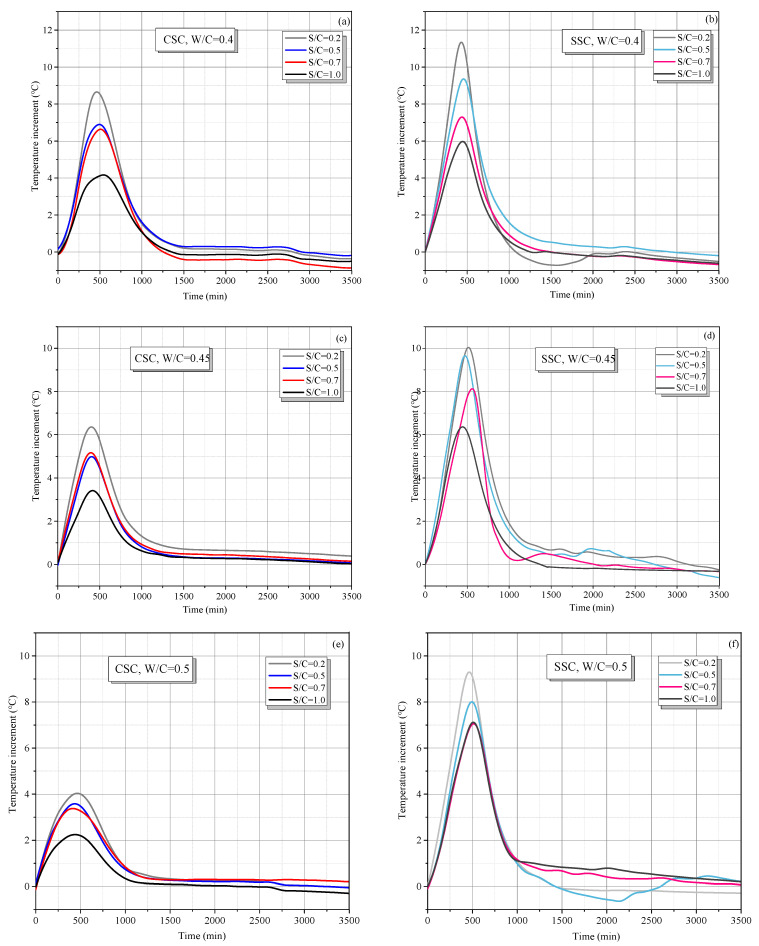

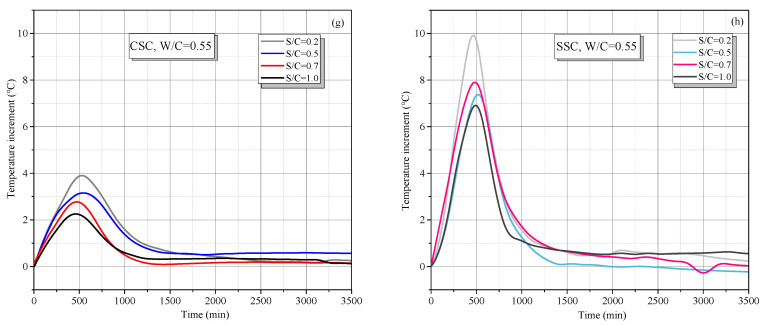
Hydration temperature increment of the CSC and SSC cement pastes under different W/C and S/C: (**a**) CSC, W/C = 0.4; (**b**) SSC, W/C = 0.4; (**c**) CSC, W/C = 0.45; (**d**) SSC, W/C = 0.45; (**e**) CSC, W/C = 0.5; (**f**) SSC, W/C = 0.5; (**g**) CSC, W/C = 0.55; (**h**) SSC, W/C = 0.55.

Temperature is an important indicator for the early-age hydration process, which reflects the hydration rate and would affect the later strength. In this study, the temperature increment with respect to room temperature (20 °C) was considered and the temperature increment curves of CSC and SSC mortar specimens with various proportions are shown in Figure 5. It can be noted that all the temperature increment curves were of a similar shape, meeting the unimodal model. The temperature variation went through two periods.

The first was the hydration stage, during which the temperature rose rapidly to the peak point. The corresponding time is defined as the initial setting time [25,38,39]. The initial setting time for CSC and SSC mortar specimens was both around 500 min (about 8 h), which seems to have little association with the proportion. The possible reason may be that the difference between specimens of different proportions was the mass of the sand and water instead of the cement content, which indicates that the initial setting time may be mainly associated with the cement content. Differently, the peak hydration temperature of CSC mortar was much lower than that of SSC mortar. Table 2 lists the percentage decrease in the peak temperature of CSC relative to SSC. The relative temperature was defined as $\frac{T_{ssc}-T_{csc}}{\left|T_{ssc}\right|}\times 100\%$, where $T_{ssc}$ and $T_{csc}$ represent the peak temperature of SSC and CSC, respectively. The maximum relative percentage decrease in the peak temperature was obtained when S/C = 1.0 and W/C = 0.5 with the value of 68.5%. It may be related to the rich internal pores of coral sand, which increased the heat capacity of CSC mortar and absorbed more heat of hydration.

In the second stage, for both SSC and CCS mortars, the relative temperature decreased and tended to reach 0 °C before 1000 min, which indicates that the early hydration mainly occurred within 1000 min and the aggregate of coral sand seems to have little impact on the time of early hydration. It should be noted that two kinds of negative temperature increments at the end of experiments sometimes occur. One is the negative temperature in Figure 5b (S/C = 0.2) and Figure 5f (S/C = 0.5) showing an unstable temperature fluctuation, and the other is the tendency of the final stable results to be negative (Figure 5a,b,d). The first phenomenon may be caused by the drift of the central wavelength of the optical fiber demodulation acquisition channel (equipment error) due to the long time running. Moreover, for the second phenomenon, the selection of the initial point of optical fiber temperature measurement may be the main reason. Specifically, even if we insert the optical fiber with a copper tube into the sample immediately after adding water and stirring, the cement still has hydration behavior in a short time, i.e., the initial temperature measured by FBG may be higher than 20 °C, therefore, when the hydration of sample is over, the temperature tends to be 20 °C (room temperature), and the relative negative temperature will appear. However, this paper mainly studies the temperature change process of early hydration rather than the specific values. The maximum negative temperature measured is no more than 1 °C, and the final temperature is stable. Considering the high sensitivity of FBG and equipment error, these results are thought to be reasonable.

**Figure 6:**
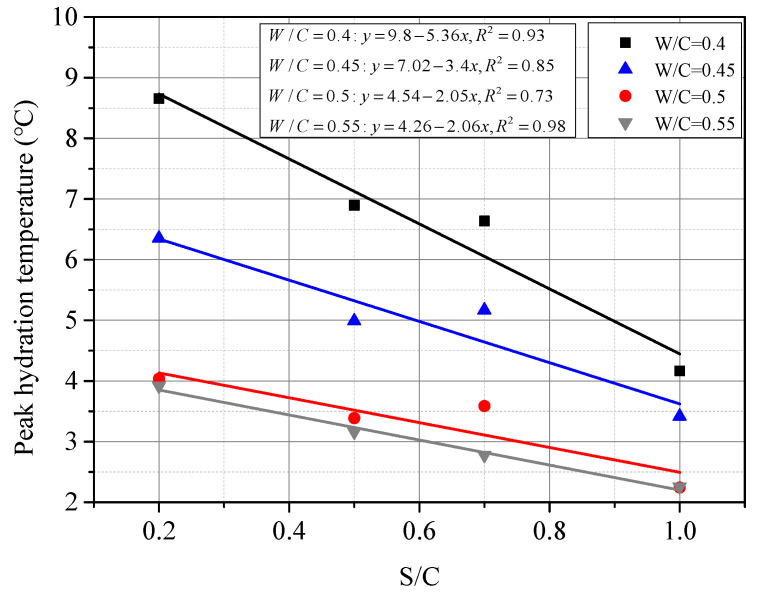
Fitted peak hydration temperature with S/C under the same W/C in CSC.

**Figure 7:**
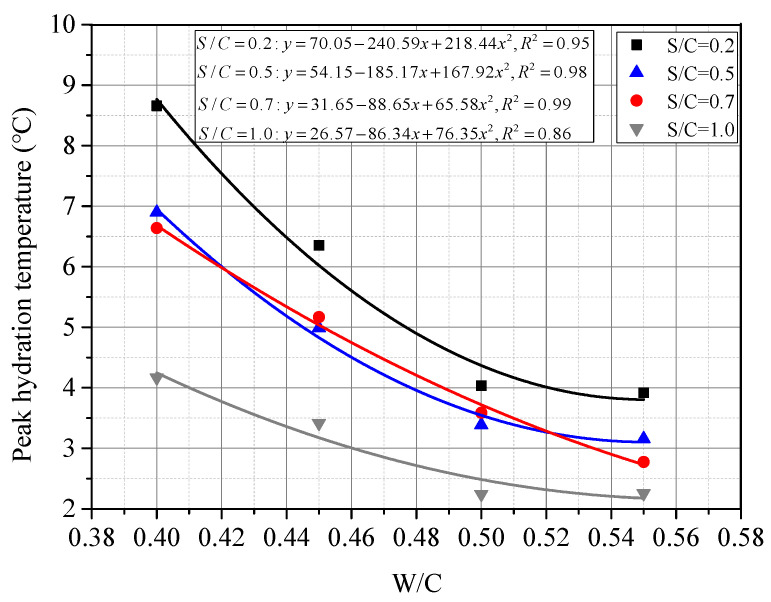
Fitted peak hydration temperature with W/C under the same S/C in CSC.

**Figure 8:**
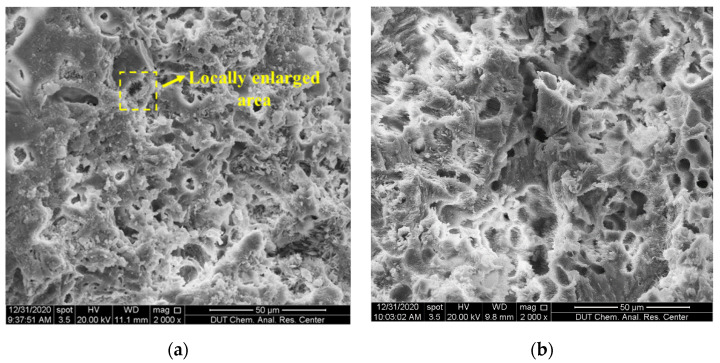

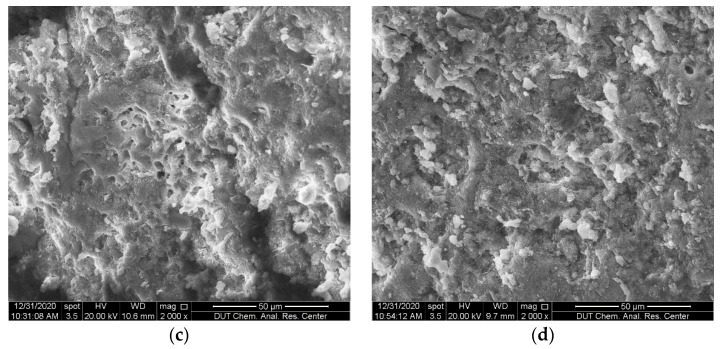
Micro-morphologies of CSC cement paste for different W/C ratios under S/C = 1.0 (2000 magnifications): (**a**) W/C = 0.40; (**b**) W/C = 0.45; (**c**) W/C = 0.50; (**d**) W/C = 0.55.

Figure 6 shows the measured peak hydration temperature and fitting functions of CSC mortar. It seems that the peak hydration temperature shows a linear downward trend along with the increase in S/C when W/C was constant, i.e., the temperature decreased with the content of coral sand. This phenomenon can also be explained by the corollary in the previous section that the increase in heat capacity due to the abundant internal pores of coral sand made the difference. As shown in Figure 7, the relationship between the peak hydration temperature and W/C was fitted by a quadratic polynomial. The temperature decreased with water content, which was also suggested to be related to the internal pores of coral sand. Coral sand particles absorbed more water when mixed with water and increased the heat capacity of the mortar, which resulted in a lower peak hydration temperature. Figure 7 also indicated that the peak hydration temperature shifted to become stable when W/C reached 0.5. It was inferred that as the W/C was higher than 0.5, the amount of water absorption by the internal pores of coral sand tends to reach the limit, and the capacity of coral sand was stabilizing. This phenomenon can be also validated by the differences of micro-morphologies between SCS mortars with various W/C, as shown in Figure 8. With the increase in W/C, it can be observed that the surface of CSC was smoother, and the porosity clearly decreased. Figure 8c,d shows little change in the porosity of CSC mortar after W/C reached 0.5.

### 3.2. Early Shrinkage Strain

**Figure 9:**
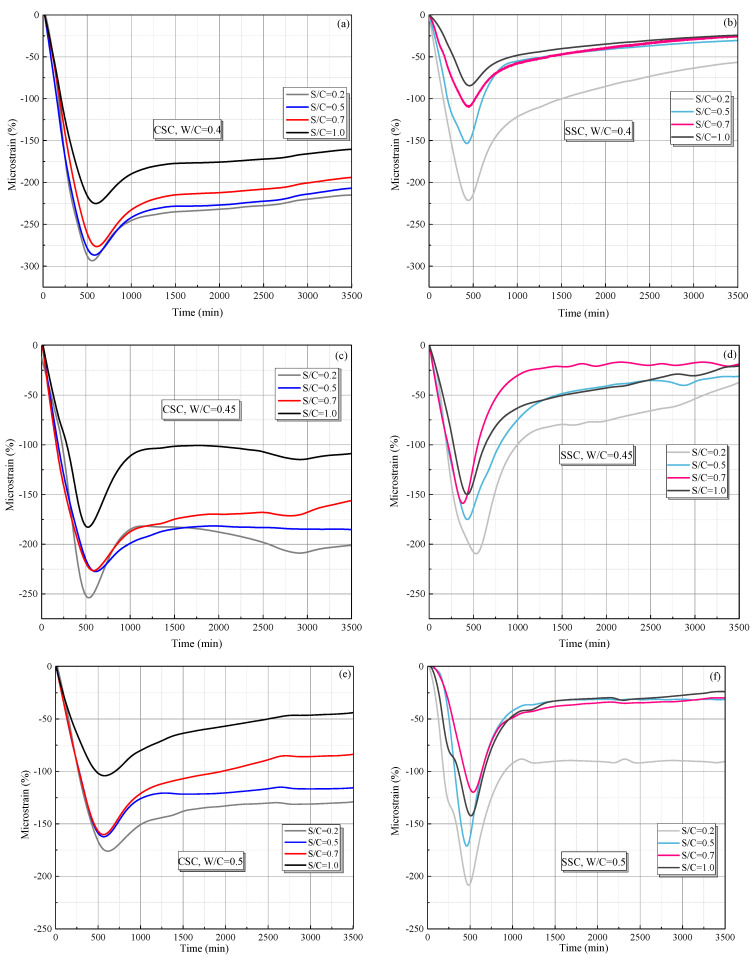

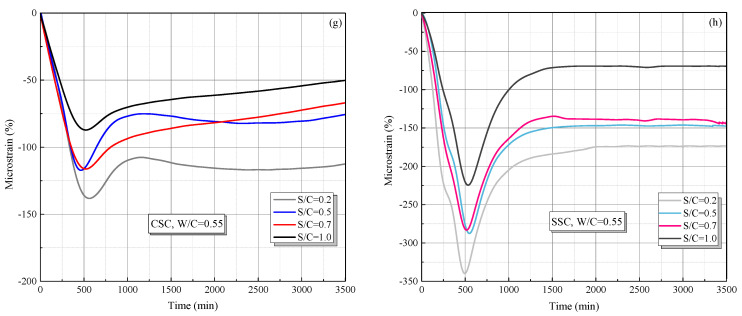
Hydration shrinkage strain of the CSC and SSC cement pastes under different W/C and S/C: (**a**) CSC, W/C = 0.4; (**b**) SSC, W/C = 0.4; (**c**) CSC, W/C = 0.45; (**d**) SSC, W/C = 0.45; (**e**) CSC, W/C = 0.5; (**f**) SSC, W/C = 0.5; (**g**) CSC, W/C = 0.55; (**h**) SSC, W/C = 0.55.

The internal shrinkage strains were obtained by FBG sensors and calculated by Equation (2). The shrinkage strain results of CSC specimens are shown in Figure 9. It can be found that the shrinkage strains were decreased and then increased with an increase hydration time. After 1000 min, the shrinkage strains were slightly varied with the hydration time. Moreover, it can be observed that the peak shrinkage of CSC was larger than that of SSC for the lower value of W/C (i.e., W/C = 0.4 or 0.45). However, when the W/C increased to a larger value (0.5, 0.55), the opposite phenomenon can be observed. The relative differences of peak micro-strain between CSC and SSC can be defined as $\frac{\varepsilon_{ss}-\varepsilon_{cs}}{\left|\varepsilon_{ss}\right|}\times 100\%$. Table 3 lists the relative difference of peak shrinkage strains between SSC (i.e., $\varepsilon_{ss}$) and CSC (i.e., $\varepsilon_{cs}$). It shows that the average peak strains of CSC were higher than that of SSC. The possible reasons for such a wide range of peak strain variation may be attributed to the following reasons.

**Figure 10:**
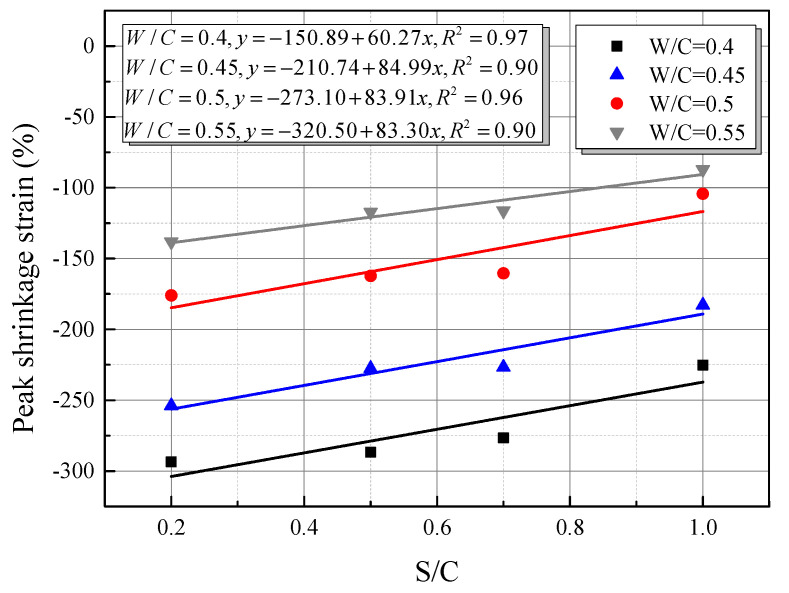
Fitted peak shrinkage strain with S/C under the same W/C in CSC mortar.

**Figure 11:**
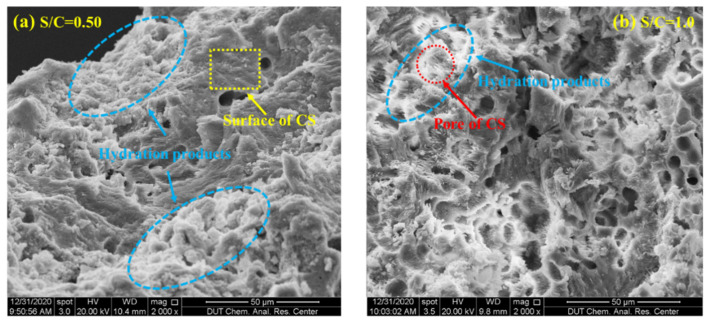
Micro-morphologies of CSC mortar for different S/C under W/C = 0.45: (**a**) S/C = 0.50; (**b**) S/C = 1.0 (2000 magnifications).

**Figure 12:**
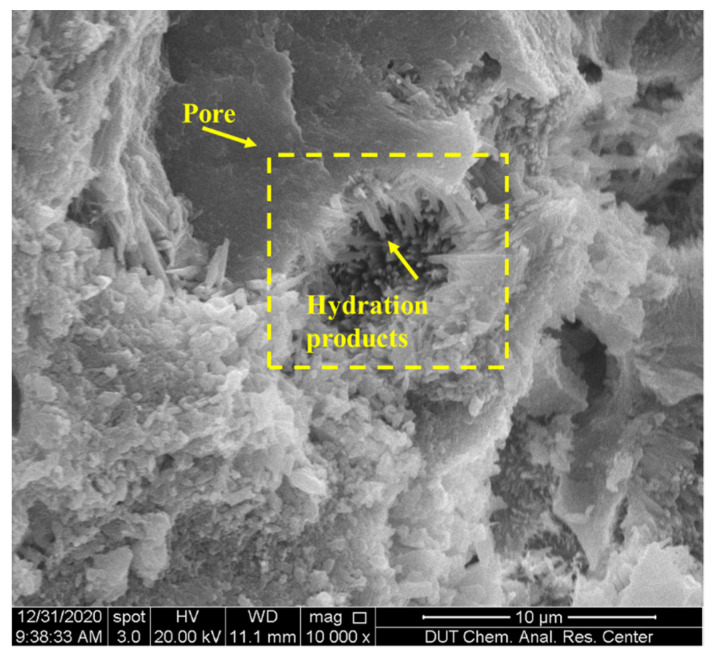
Locally enlarged micro-morphologies of CSC cement paste for S/C = 1.0 and W/C = 0.40 (10,000 magnifications).

(1) When the W/C is small, there is less free water in the cement paste and it has little impact on the shrinkage, thus the peak strain is mainly affected by the aggregate. The existence of aggregate can inhibit the shrinkage of hydration products, which is proven in Figure 10, i.e., with the increase in aggregate material (S/C), the peak shrinkage strain in CSC decreases linearly. Meanwhile, a larger aggregate modulus can also inhibit the shrinkage of hydration products. Due to the porosity of CS, its aggregate modulus is lower than that of SS, therefore leading to a relatively serious peak strain in CSC.
(2) When the W/C is large, there is more free water in the cement paste, therefore the autogenous shrinkage of cement is enhanced due to more water loss (shown in SS). However, because CS has good water absorption and releasing characteristics [40], it can absorb excess free water in the case of large W/C, leading to a reduction of water loss.
(3) Figure 11 shows the micro-morphologies of CS cement paste for different S/C, and it shows that the hydration products are mainly adsorbed on the surface of CS at a low S/C. Meanwhile, with the increase in S/C, more products are found in pores, which is proven in a locally enlarged micro-morphology in Figure 12. Fibrous hydration products in the pores mean the wet environment formed in the pores can cure the cement products and further reduce the shrinkage strain. Moreover, due to the uneven surface of CS, hydration products can be closely combined with CS to reduce a part of the volume shrinkage.

## 4. Conclusions

The early-hydration behavior of coral sand mortar is an important factor for the mechanical properties of cement blocks in later stages. In order to obtain continuous hydration temperature and internal shrinkage strain measurements in specimens with lower strength, this study presented a novel monitoring system based on FBG for early-age hydration monitoring. A total of 32 specimens with different mix proportions were prepared for both CSC and SSC to evaluate the impact of W/C and S/C on the early-age hydration behavior. Moreover, the micro mechanism of CSC was also analyzed through the micro-morphology by SEM results. Based on the obtained results, some conclusions can be drawn:

(1) The early-age hydration temperatures and shrinkage strains of cement paste can be measured simultaneously by the FBG monitoring system with high accuracy and stability. Due to the high sensitivity and fitness of optical fiber, the proposed monitoring system provides a good measurement method for studying the early hydration behavior of cement paste. The results show that the W/C and S/C have less of an effect on the initial setting time of CSC mortar. The peak strain and temperature of CSC mortar vary monotonically with W/C and S/C.
(2) Compared with the SSC, the peak hydration temperature of CSC is significantly reduced. The maximum relative difference of the peak temperature was 68.5%, which indicated that the CSC can effectively reduce the influence of hydration heat due to better heat-absorption capacity. As an eco-friendly lightweight aggregate, coral sand can effectively alleviate the hydration heat of mass concrete.
(3) With the increase in S/C, the hydration peak temperature of CSC decreased linearly, which indicated that CSC had superior heat-absorption capacity due to the porosity. The peak temperature decreased gradually with the increase in W/C, indicating that CSC can absorb excess water and further improve its heat-absorption capacity with the increase in W/C.
(4) Due to the relatively small modulus, the peak shrinkage strain of CSC was larger than that of SSC for a smaller value of W/C (i.e., 0.4 or 0.45), while, with the increase in W/C (0.5, 0.55), more free water evaporation leads to serious shrinkage strain in SSC, and a better water retention ability reduces the shrinkage in CSC due to its porosity characteristics of CS. Therefore, CS is more suitable as an aggregate than SS for the design of high W/C under the meeting of strength.

## Figures and Tables

**Table 1:** The proportion of CSC and SSC mortars.

Test Number	OPC/g	W/C	Water/g	Aggregate/g	S/C
1	80	0.4	32	16	0.2
2				40	0.5
3				56	0.7
4				80	1
5	80	0.45	36	16	0.2
6				40	0.5
7				56	0.7
8				80	1
9	80	0.5	40	16	0.2
10				40	0.5
11				56	0.7
12				80	1
13	80	0.55	44	16	0.2
14				40	0.5
15				56	0.7
16				80	1

**Table 2:** Percentage decrease in peak temperature of CSC relative to SSC.

S/C	Peak Temperature Difference (%)			
	W/C = 0.4	W/C = 0.45	W/C = 0.5	W/C = 0.55
0.2	23.7	36.7	56.6	60.5
0.5	26.3	48.3	57.7	57.2
0.7	9.0	36.4	49.2	64.9
1	30.3	46.3	68.5	67.4

**Table 3:** Percentage decrease in peak shrinkage strains of CSC relative to SSC.

W/C	Relative Difference of Peak Shrinkage Strain (%)				
	S/C = 0.2	S/C = 0.2	S/C = 0.2	S/C = 0.1	Mean Value
0.4	32.52	86.98	151.20	166.77	109.37
0.45	21.18	29.93	42.67	21.97	28.94
0.5	−15.56	−5.20	33.92	−26.71	−3.39
0.55	−59.33	−59.22	−58.94	−61.17	−59.67

## Data Availability

Not applicable.
